# An analysis of the mediating influence of depression on the association between early-life caregiver relationships and cognitive function: a cohort study based on the CHARLS database

**DOI:** 10.3389/fpsyt.2025.1555336

**Published:** 2025-03-04

**Authors:** Jingkai He, Hui Zhang, Zhuocheng Wu, Liuyin Jin, Yunxin Ji

**Affiliations:** ^1^ Department of Psychosomatic Medicine, The First Affiliated Hospital of Ningbo University, Zhejiang Regional Medical Center, Ningbo, Zhejiang, China; ^2^ Third Ward, Ningbo Kangning Hospital, Ningbo, Zhejiang, China; ^3^ Faculty of Clinical Medicine, Southern Medical University, Guangzhou, Guangdong, China; ^4^ Science and Education Section, Lishui Second People’s Hospital, Lishui, China

**Keywords:** cognitive impairment, caregiver relationships, depression, mediating effect, gender differences, CHARLS

## Abstract

**Objective:**

This study aims to elucidate the potential mediating role of depression in the relationship between early-life relationships with caregivers and subsequent cognitive impairment in later adulthood.

**Methods:**

Leveraging data from the China Health and Retirement Longitudinal Study (CHARLS), we included a cohort of 10,828 participants aged 45 and above. We assessed the quality of childhood caregiver relationships using specific relationship scores and evaluated cognitive function through the Mini-Mental State Examination (MMSE) scores obtained in 2018. Depressive symptoms were measured utilizing the CES-D-10 scale. To explore the interrelationships among these variables, we employed multivariable logistic regression models and non-parametric bootstrap methods to assess the mediating effect of depression.

**Results:**

The study unveiled significant disparities between the cognitive impairment group and the cognitively normal group in terms of gender, age, educational attainment, hypertension status, depression levels, and the nature of relationships with parents during childhood. Regression analyses demonstrated a positive correlation between childhood caregiver relationship scores and cognitive impairment (Odds Ratio [OR] = 1.01, 95% Confidence Interval [CI]: 1.00–1.02, *p* = 0.01). Importantly, depression exhibited a significant mediating effect in this association, accounting for approximately 20% of the total effect (Proportion Mediated = 20%, *p* = 0.008). The influence was more pronounced concerning relationships with female caregivers, where depression mediated 11.5% of the effect (Proportion Mediated = 11.5%, *p* < 0.001). Conversely, the mediating effect of depression on relationships with male caregivers was not statistically significant.

**Conclusion:**

The findings underscore that early-life relationships with caregivers have a profound impact on cognitive function in later life, with depression serving as a crucial mediator, particularly among women. These insights highlight the importance of fostering a positive familial environment during childhood, mitigating adverse parenting practices, and implementing early interventions targeting depression to potentially reduce the risk of cognitive impairment and promote healthy aging.

## 1 Introduction

With the rapid aging of the global population, dementia is affecting an ever-growing number of individuals. Currently, approximately 50 million people suffer from dementia, and this figure is projected to rise to 152 million by 2050 (1). In particular, China, which has the world’s largest population of older adults, has 267 million people aged 60 and above, and the prevalence of dementia stands at 5.14% (2). This not only severely compromises the quality of life and social functioning of older adults but also imposes a heavy burden on families and society (3, 4).The early-life environment plays a pivotal role in an individual’s brain development. Factors that influence early childhood development include parental education level, maternal mental health, malnutrition, ethnicity, family environment characteristics, the quality of child care, parent-child interactions, and sociocultural background (5). Within the family environment, elements such as the parent-child relationship, domestic violence, emotional abuse or neglect, physical neglect, and physical abuse can all affect a child’s development, thereby influencing both physical and mental health later in life. Conversely, a positive family environment confers lifelong benefits (6–8).Multiple studies have demonstrated that childhood experiences of physical abuse and emotional neglect are associated with an increased risk of depression and are closely linked to a decline in cognitive function (9–11). However, research on the specific interrelationships among parent-child relationships, depression, and cognition remains relatively scarce. Investigating the mediating role of depression in these associations is therefore of considerable importance for understanding the onset and progression of cognitive impairment and for enhancing cognitive health in older adults.

China Health and Retirement Longitudinal Study (CHARLS) encompasses an extensive assortment of demographic, socioeconomic, health, and mental health indicators, including rich information on early-life parent-child relationships, depressive symptoms (assessed via the CES-D scale), and cognitive performance (e.g., memory and calculation skills). Leveraging these data within a Chinese cultural framework enables a rigorous examination of how childhood relationships with parents, in conjunction with depression, might exert a lasting impact on cognitive function, thereby offering robust empirical support for the development of targeted interventions in China’s aging population (12).Through an in-depth analysis of the CHARLS dataset, this study endeavors to elucidate how early-life dynamics with parental figures may indirectly mold cognitive abilities through the pathway of depressive symptoms, thereby revealing an essential mediating mechanism. The findings will not only provide fresh insights into the multifaceted etiology of cognitive impairment but also lay a solid foundation for identifying particularly vulnerable demographic groups. Moreover, these results serve as a crucial evidentiary basis for crafting and refining public health strategies aimed at fostering healthy aging, underlining both the theoretical and practical significance of this line of inquiry.

## 2 Methods

### 2.1 Study population

**Figure 1:**
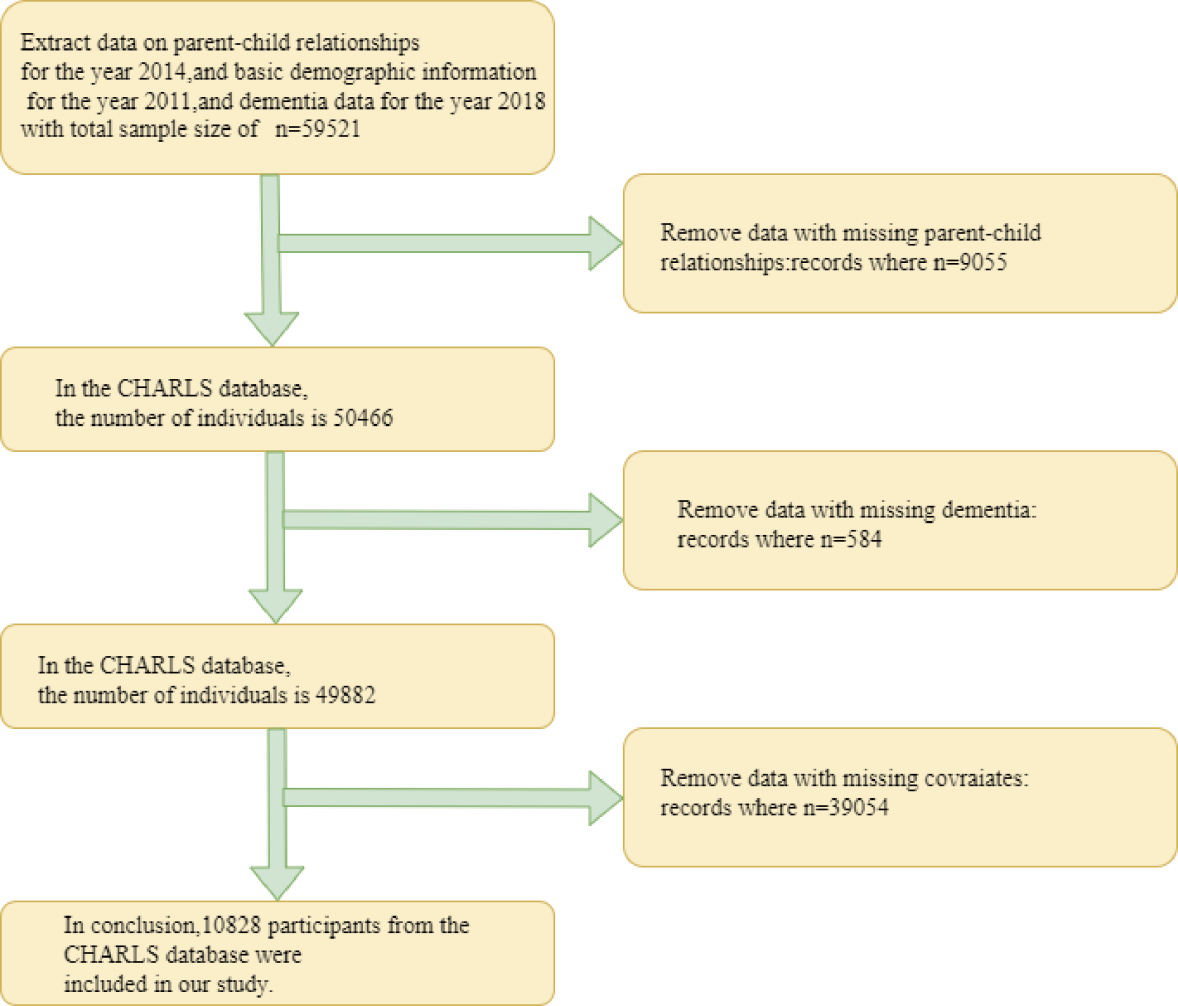
Data selection flowchart.

This investigation leveraged data from the China Health and Retirement Longitudinal Study (CHARLS), a nationally representative longitudinal survey of Chinese residents aged 45 years and older that captures a wide array of demographic, socioeconomic, health, and retirement-related information (13).To examine the potential impact of early parent-child relationships and depressive symptoms on late-life cognitive impairment, we conducted a systematic data extraction and filtering process. Initially, data were extracted from the CHARLS database, including the 2014 parent-child relationship questionnaire, baseline demographic and covariate information from 2011 (e.g., gender, age, residence, education, ethnicity, marital status, smoking, drinking, BMI, obesity, hypertension, and diabetes), and dementia assessment data from 2018. The total sample size was 59,521 participants. We then applied a series of exclusion steps. First, 9,055 participants with missing parent-child relationship data were removed, resulting in 50,466 individuals. Next, 584 participants with missing dementia data were excluded, reducing the sample to 49,882. Finally, 39,054 participants with missing baseline covariate data were excluded, leaving a final sample of 10,828 participants eligible for the analysis. A detailed flowchart of the sample selection process is presented in **Figure 1**.

### 2.2 Cognitive function assessment

The 2018 wave of CHARLS administered the Chinese version of MMSE, which served as the primary outcome measure for the present study. This evaluation targeted two key domains of cognitive function—episodic memory and mental integrity. We generated an overall cognitive score spanning from 0 to 31 by summing the scores from these two domains, with higher totals indicating more robust cognitive performance. For the purposes of this investigation, individuals with a total cognitive score below 11 were classified as having cognitive impairment, whereas those scoring 11 or above were deemed cognitively intact (14).

### 2.3 Assessment of depression

The CHARLS database deploys the 10-item Center for Epidemiologic Studies Depression Scale (CES-D-10) to gauge depressive symptoms (15). This succinct yet robust measure has been extensively utilized for assessing depression in older adult populations, and its psychometric soundness—particularly its reliability and validity—has been well established among Chinese older adults. The CES-D-10 encompasses 10 items, each rated on a four-point scale: (1) rarely or almost never (<1 day), (2) sometimes (1–2 days), (3) occasionally (3–4 days), and (4) most or all of the time (5–7 days). Each item corresponds to a score ranging from 0 to 3, culminating in a total score from 0 to 30; lower scores indicate fewer depressive symptoms. According to widely recognized criteria, individuals with scores of 10 or above are regarded as having significant depressive symptoms (16, 17).

### 2.4 Assessment of early-life relationships with caregivers

First, we extracted data from individuals who participated in the 2014 Parent-Child Relationship Questionnaire, a component of CHARLS. This large-scale study investigates various aspects of health, socioeconomic status, and family relationships among middle-aged and older adults in China. The Parent-Child Relationship Questionnaire specifically collected retrospective information about participants’ relationships with their parents during childhood and adulthood, focusing on dimensions such as interactions, support, and conflicts.

The questionnaire was administered to individuals aged 45 and older who participated in the 2014 wave of CHARLS. The initial sample consisted of approximately 17,000 participants, representing a diverse population from urban and rural areas, with varying levels of education and socioeconomic backgrounds. By examining emotional bonds, parental support behaviors, parenting styles, financial assistance, and family conflicts, the questionnaire provides valuable data for exploring the long-term impact of parent-child relationships on health and psychological well-being later in life (18).

From this instrument, we selected 12 items to gauge participants’ early-life interactions with their caregivers. The response options were scored sequentially (e.g., a response of “1” received 1 point, “2” received 2 points, and so forth), and items 4, 5, 6, 7, 9, 10, 11, and 12 were not reverse-coded. These scores were then aggregated to generate a composite index of the early-life caregiver relationship, where higher totals signaled a more adverse relationship. In addition to calculating a unified index, we separately computed total scores for male and female caregivers. We then carried out additional analyses to determine how childhood relationships with each caregiver type (female versus male) might shape depressive outcomes later in adulthood. Further details on the specific items can be found in the **Supplementary File**.

### 2.5 Covariate assessment

This study chiefly examines the associations among caregiver-child relationships, depression, and cognitive impairment. The onset of cognitive impairment is intricately linked to a range of risk factors, including sex, age, residential location, educational attainment, hypertension, diabetes, ethnicity, and obesity (12). Accordingly, the following variables were incorporated as covariates in our analyses: sex, age, residential location, educational level, ethnicity, marital status, smoking, alcohol use, BMI, obesity, hypertension, and diabetes (19).

### 2.6 Statistical analysis

In this study, continuous variables were summarized as the mean ± standard deviation (SD) or median (interquartile range, IQR), whereas categorical variables were reported as percentages. To elucidate the associations among cognitive function, caregiver-child relationships, and depression, we employed multivariable logistic regression models to calculate odds ratios (OR) and 95% confidence intervals (CI). Three models were constructed: Model 1: Unadjusted (crude) model. Model 2: Adjusted for fundamental demographic characteristics (i.e., age, sex, educational level, place of residence, marital status, and ethnicity).Model 3: Further adjusted for additional covariates (i.e., smoking, alcohol use, hypertension, diabetes, body mass index [BMI], and obesity).

Subgroup analyses were subsequently performed to explore the nuances of how caregiver-child relationships and depression interact to influence cognitive outcomes across different demographic strata. To provide a more comprehensive understanding of the mediating role of depression in the relationship between total scores and cognitive function, we employed a causal mediation framework. Initially, we constructed mediation and outcome models without incorporating covariates, with the aim of evaluating both the direct and indirect effects of total scores and depression on cognitive function, as well as the impact of total scores on depression. We then reconstructed these models after adjusting for all pertinent covariates, thus offering a more robust validation of the pathway through which total scores may affect cognitive function via depression.

We utilized a nonparametric bootstrap procedure (5,000 simulations) to estimate the mediation effect, direct effect, total effect, and the proportion of the total effect explained by mediation, along with the corresponding 95% confidence intervals and p-values. All data analyses were conducted using R version 4.3.2. The mediation package was employed for the causal mediation analysis, thereby yielding essential insights into the underlying causal pathways among the study variables.

## 3 Results

### 3.1 Basic information of participants

**Table 1** provides an overview of the demographic and clinical characteristics of the study cohort. Altogether, 10,828 participants were enrolled, of whom 6,138 (56.67%) were classified as cognitively normal, and 4,690 (43.33%) exhibited cognitive impairment. With respect to gender distribution, females constituted 67.02% of the overall sample and males 32.98%. Notably, the proportion of females was significantly higher in the cognitive impairment group compared with the cognitively normal group (75.54% vs. 60.51%, *p* < 0.0001).An examination of age revealed that individuals in the cognitive impairment group had a mean age of 60.35 ± 8.21 years, which was markedly older than those in the cognitively normal group (55.54 ± 7.65 years, *p* < 0.0001). Regarding marital status, the prevalence of marriage exceeded 98.73% in both groups, showing no significant difference (*p* = 0.47). Significant differences were observed in education levels (p < 0.0001). The proportion of illiterate individuals was markedly higher in the cognitive impairment group compared to the cognitively normal group (53.77% vs. 8.44%). Additionally, the number of individuals with education beyond middle school was lower in the cognitive impairment group than in the cognitively normal group. Geographical location also emerged as an influential factor: 75.31% of individuals in the cognitive impairment group were rural residents, significantly surpassing the 58.36% recorded in the cognitively normal group (*p* < 0.0001). Regarding ethnicity, the vast majority (92.92%) were Han Chinese, and no significant ethnic differences were observed between the two groups (*p* = 0.69).Analysis of lifestyle factors and health status revealed that the smoking rate was notably lower in the cognitive impairment group (18.32%) relative to the cognitively normal group (22.42%, *p* < 0.0001). Moreover, alcohol consumption varied significantly (*p* = 0.01), with fewer drinkers in the cognitive impairment group. Hypertension prevalence was significantly elevated in the cognitive impairment group (37.42% vs. 30.37%, *p* < 0.0001), although diabetes prevalence did not differ (*p* = 0.52).Body mass index (BMI) was also significantly lower in the cognitive impairment group (23.45 ± 3.76 vs. 24.37 ± 3.91, *p* < 0.0001). As for weight classification, the cognitive impairment group contained a higher proportion of underweight individuals (7.53% vs. 3.88%) but a lower proportion of obese individuals (11.30% vs. 15.30%, *p* < 0.0001).Although no significant difference was detected in the overall total_score (*p* = 0.69), the female_total_score was slightly higher in the cognitive impairment group than in the cognitively normal group (12.74 ± 3.59 vs. 12.59 ± 3.33, *p* = 0.04). Conversely, the male_total_score was marginally lower in the cognitive impairment group (8.88 ± 2.56 vs. 9.06 ± 2.41, *p* < 0.001).In sum, key variables—such as gender, age, educational background, hypertension, depression, and caregiver relationships—differed significantly between the cognitive impairment and cognitively normal groups. These findings underscore the potential importance of these factors in influencing cognitive impairment.

**Table 1:** Basic Information.

Variable	Total (n=10828)	No (n=6138)	Yes (n=4690)	Statistic	P.value
sex				271.23	<0.0001
female	7257(67.02)	3714(60.51)	3543(75.54)		
male	3571(32.98)	2424(39.49)	1147(24.46)		
**age**	57.62 ± 8.25	55.54 ± 7.65	60.35 ± 8.21	-31.12	**<0.0001**
marital_status				0.52	0.47
married	10691(98.73)	6065(98.81)	4626(98.64)		
no married	137( 1.27)	73( 1.19)	64( 1.36)		
education				3407.43	<0.0001
illiterate	3040(28.08)	518( 8.44)	2522(53.77)		
middle school	2996(27.67)	2659(43.32)	337( 7.19)		
primary school	4452(41.12)	2641(43.03)	1811(38.61)		
university	340( 3.14)	320( 5.21)	20( 0.43)		
location				338.25	<0.0001
rural	7114(65.70)	3582(58.36)	3532(75.31)		
urban	3714(34.30)	2556(41.64)	1158(24.69)		
nationality				0.16	0.69
han	10061(92.92)	5709(93.01)	4352(92.79)		
minority	767( 7.08)	429( 6.99)	338( 7.21)		
smoke				27.06	<0.0001
no smoke	8593(79.36)	4762(77.58)	3831(81.68)		
smoke	2235(20.64)	1376(22.42)	859(18.32)		
drink				8.63	0.01
<1ml	1205(11.13)	730(11.89)	475(10.13)		
>1ml	1086(10.03)	618(10.07)	468( 9.98)		
no	8537(78.84)	4790(78.04)	3747(79.89)		
hypertension				59.10	<0.0001
no	7209(66.58)	4274(69.63)	2935(62.58)		
yes	3619(33.42)	1864(30.37)	1755(37.42)		
DM				0.41	0.52
no	9311(85.99)	5290(86.18)	4021(85.74)		
yes	1517(14.01)	848(13.82)	669(14.26)		
**BMI**	23.97 ± 3.87	24.37 ± 3.91	23.45 ± 3.76	12.45	**<0.0001**
obesity				145.66	<0.0001
low weight	591( 5.46)	238( 3.88)	353( 7.53)		
normal	5242(48.41)	2806(45.72)	2436(51.94)		
obesity	1469(13.57)	939(15.30)	530(11.30)		
over weight	3526(32.56)	2155(35.11)	1371(29.23)		
Depression					
depression	4076(37.64)	1934(31.51)	2142(45.67)		
normal	6752(62.36)	4204(68.49)	2548(54.33)		
**total_score**	21.64 ± 5.23	21.65 ± 5.05	21.61 ± 5.45	0.40	0.69
**female_total_score**	12.66 ± 3.44	12.59 ± 3.33	12.74 ± 3.59	-2.10	**0.04**
**male_total_score**	8.98 ± 2.48	9.06 ± 2.41	8.88 ± 2.56	3.77	**<0.001**

All p-values provided in this table are bold for visibility.

### 3.2 Logistic regression analysis of childhood caregiver relationships, depression, and cognitive impairment

**Figure 2:**
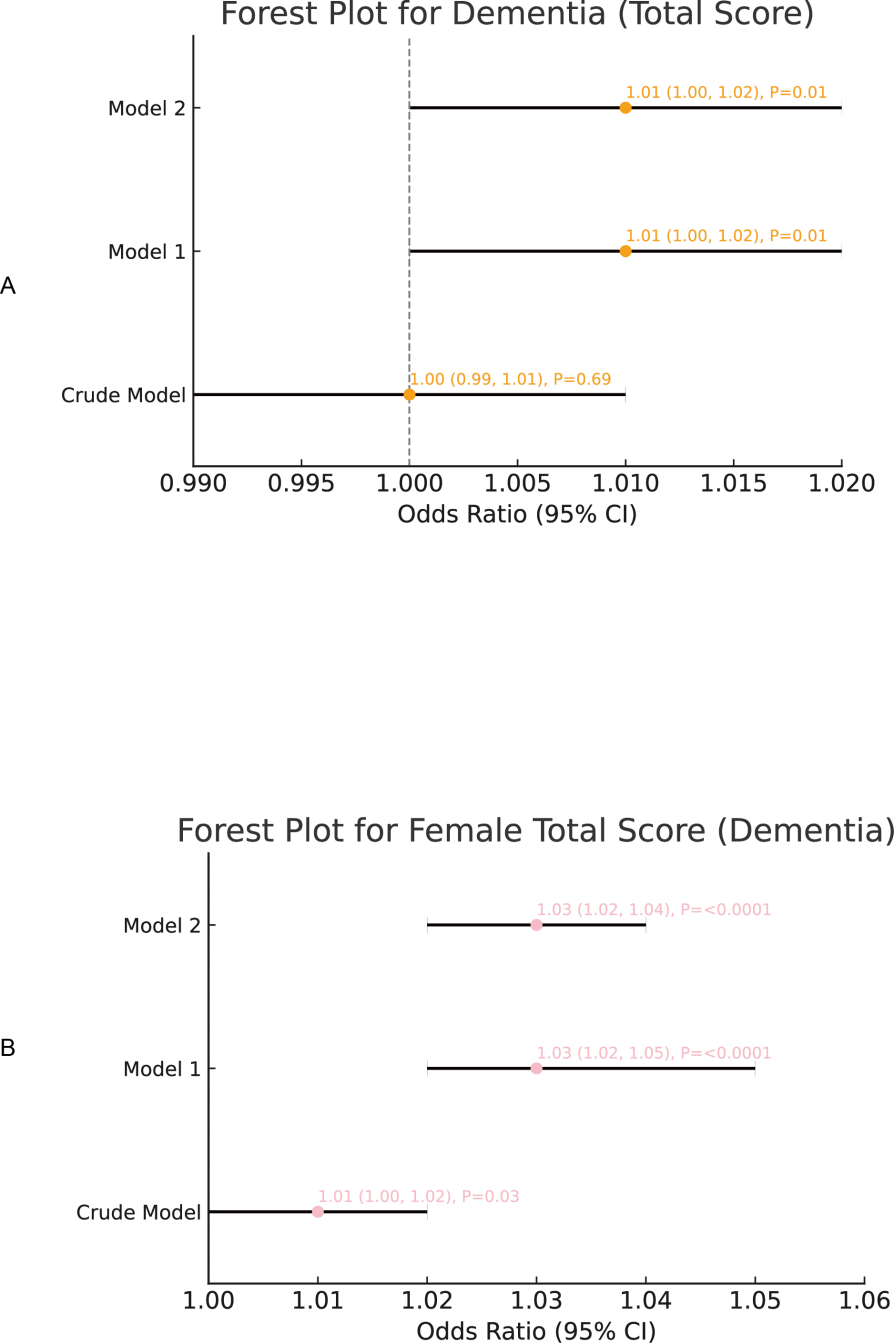
The regression analysis of caregiver relationship scores (overall, female), and the risk of cognitive impairment. **(A)** Association between total caregiver relationship score and depression and the risk of cognitive impairment. **(B)** Association between female caregiver relationship score and cognitive impairment.

**Figure 3:**
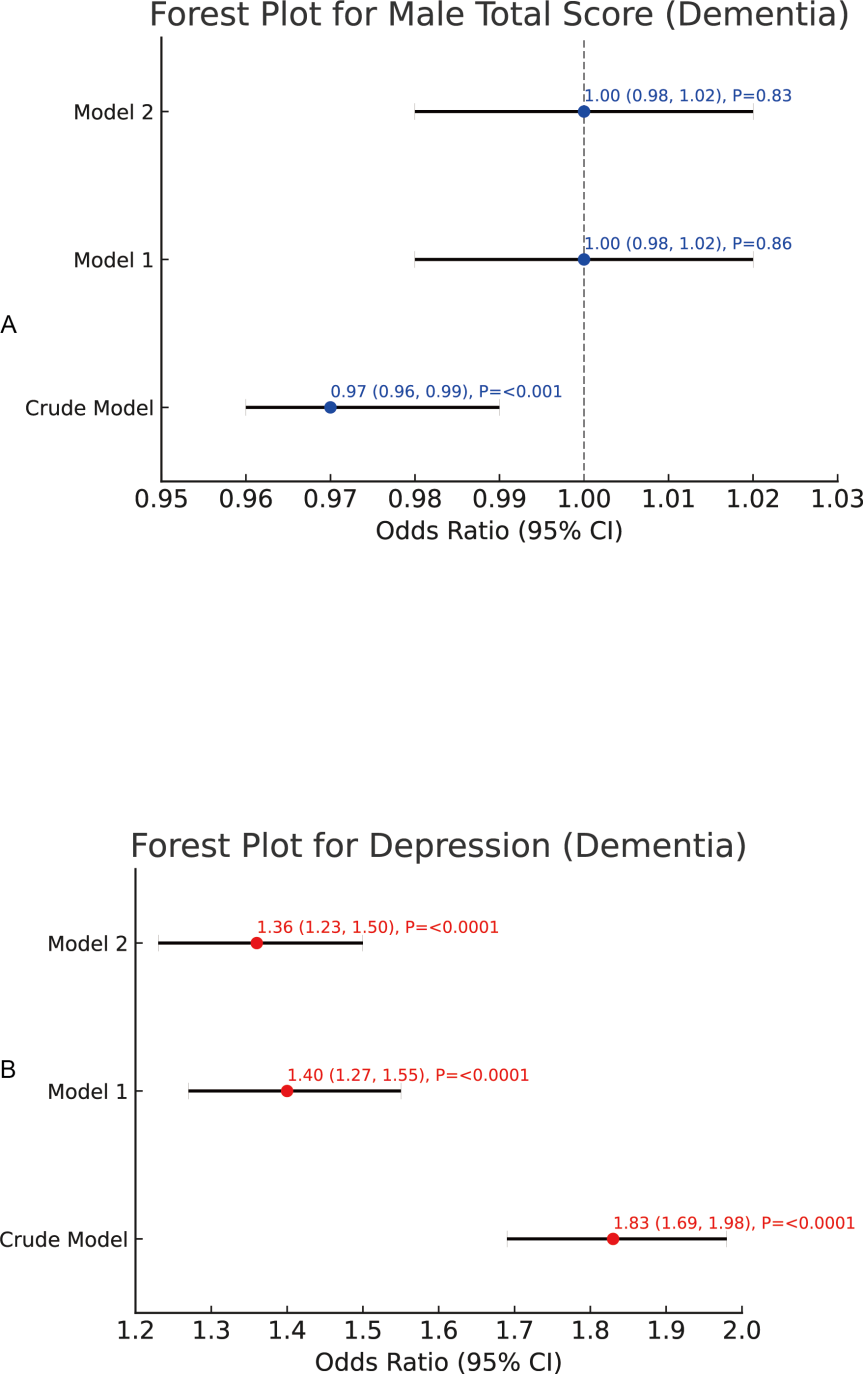
The regression analysis of male caregiver relationship scores and depression and the risk of cognitive impairment. **(A)** Association between male caregiver relationship score and depression and the risk of cognitive impairment. **(B)** Association between depression and cognitive impairment.

We conducted logistic regression analyses to elucidate the interconnections among early-life caregiver relationships, depression, and cognitive impairment. In the unadjusted model, the overall total score did not exhibit a significant association with cognitive impairment (OR = 1.00, 95% CI: 0.99–1.01, *p* = 0.69). However, upon adjusting for relevant covariates (Model 1 and Model 2), the total score demonstrated a statistically significant positive correlation with cognitive impairment (OR = 1.01, 95% CI: 1.00–1.02, *p* = 0.01).As for the female total score, the unadjusted model revealed a notable positive association with cognitive impairment (OR = 1.01, 95% CI: 1.00–1.02, *p* = 0.03). This relationship was further reinforced and remained significant after covariate adjustment (OR = 1.03, 95% CI: 1.02–1.04, *p* < 0.0001).By contrast, the male total score presented a significant negative association with cognitive impairment in the unadjusted model (OR = 0.97, 95% CI: 0.96–0.99, *p* < 0.001). Nevertheless, this inverse relationship lost significance once the covariates were taken into account (Model 1 and Model 2).Depression consistently exhibited a robust positive correlation with cognitive impairment across all models. In the unadjusted analysis, the odds ratio was 1.83 (95% CI: 1.69–1.98, *p* < 0.0001). Although the magnitude of this association tapered somewhat following covariate adjustment, it remained statistically significant (Model 2: OR = 1.36, 95% CI: 1.23–1.50, *p* < 0.0001).In summary, the total score, female total score, and depression emerged as significant predictors of cognitive impairment, whereas the male total score did not exert a notable effect after controlling for the specified covariates. Further details and visual representations of these findings can be found in **Figures 2**, **3**.

### 3.3 Subgroup analysis of childhood caregiver relationships, depression, and cognitive impairment

In the subgroup analysis of total scores and dementia, we examined variables such as gender, marital status, education level, residence, ethnicity, smoking, alcohol consumption, hypertension, diabetes, and obesity. Among these, demographic factors such as gender, marital status, frequency of alcohol consumption, smoking, and weight showed limited or statistically insignificant effects. However, education level displayed significant differences across subgroups (interaction p = 0.001). Illiterate individuals had significantly higher scores (OR = 1.021, p = 0.017), whereas those who completed middle school had significantly lower scores (OR = 0.966, p = 0.004). Hypertension also showed a significant interaction effect (p = 0.006), with hypertensive individuals scoring lower than non-hypertensive individuals (OR = 0.985, p = 0.02). For more details, please refer to **Supplementary Table S1** in the attached document (attachment.docx).

In the subgroup analysis of the caregiver relationship total scores and dementia for female caregivers, we found that scores were significantly higher for participants who were illiterate or had only completed primary school, while those with middle school education had significantly lower scores. Additionally, participants without hypertension scored higher than those with hypertension. Smoking and low-frequency alcohol consumption (e.g., occasional drinking, such as monthly) were also associated with higher scores. Notably, participants with normal weight scored higher than those who were underweight, overweight, or obese. Further stratification by gender and marital status indicated that males and married individuals scored significantly higher than females and unmarried individuals. For more details, please refer to **Supplementary Table S2** in the attached document (attachment.docx).

In the subgroup analysis of the caregiver relationship total scores and dementia for male caregivers, we found that married men scored significantly lower, while the scores of unmarried men approached statistical significance. Men with middle school education also had significantly lower scores, whereas no significant differences were observed among other education subgroups. Additionally, both rural and urban male residents scored significantly lower, and Han Chinese men scored significantly lower than ethnic minority men, though the results for minority men did not reach statistical significance. Regarding health-related variables, hypertensive men scored significantly lower, while no meaningful differences were observed among non-hypertensive men. Non-smoking and non-drinking men also scored significantly lower, though the results for smoking and drinking groups did not reach statistical significance. Underweight men had significantly lower scores, while those with normal weight and overweight scores were slightly below statistical significance, and no significant differences were observed in the obese group. Furthermore, men without diabetes scored significantly lower, while the scores for men with diabetes approached significance. For more details, please refer to **Supplementary Table S3** in the attached document (attachment.docx).

In a dedicated subgroup analysis, we investigated the impact of depression on dementia across various demographic and parent-child relationship subgroups. Depression was consistently associated with a significantly increased risk of dementia. Specifically, both female (OR = 1.76) and male (OR = 1.62) patients showed significantly elevated risks. Married individuals, as well as those who were illiterate or had completed only primary, middle, or even higher education, were also at higher risk. Notably, ethnic minorities faced greater risks compared to Han Chinese participants. Furthermore, non-smokers and those who consumed small amounts of alcohol monthly exhibited higher risks. Compared to individuals with normal weight, overweight, or obesity, underweight individuals faced a markedly higher risk. Although hypertension and diabetes were associated with an overall increase in risk, the magnitude of this increase was not significantly different from those without these conditions. For more details, please refer to **Supplementary Table S4** in the attached document (attachment.docx).

### 3.4 The Relationship between childhood caregiver relationships and cognitive impairment—with depression as a mediator

In our mediation analyses, we employed a non-parametric bootstrap methodology with 5,000 iterations. We began by conducting mediation analyses without controlling for any additional variables and then repeated these analyses while adjusting for pertinent covariates, including age, gender, marital status, educational level, place of residence, ethnicity, smoking, alcohol consumption, hypertension, diabetes, BMI, and obesity. The key findings are outlined below:

### 3.5 Total score, depression, and dementia

Our results indicate that, in the link between total score and dementia, the indirect effect of total score via depression was highly significant. For both the control and treatment groups, the estimates were 0.001077 and 0.001076 (95% CI: 0.000797 to 0.000796, p < p < 0.01). Likewise, the average ACME (Average Causal Mediation Effect) was significant (0.001076, 95% CI: 0.000796 to 0.000796, p < 0.01), underscoring the pivotal role that depression plays in mediating the relationship between total score and cognitive impairment. By contrast, the direct effect of total score on cognitive impairment did not reach statistical significance (mean ADE [Average Direct Effect] = -0.001468, 95% CI: -0.003369 to 0, p = 0.096), nor did the total effect (estimate = -0.000392, 95% CI: -0.002257 to 0, p = 0.652). Furthermore, other potential mediators failed to achieve significance (mean estimate = -2.748572, 95% CI: -17.703305 to 15, p = 0.652). Collectively, these findings suggest that while depression is an important mediator, the direct influence of total score on cognitive impairment and its broader implications warrant further exploration. For additional details, please consult **Table 2**.

**Table 2:** Mediation analysis of caregiver's total score and cognitive impairment with depression as a mediator.

Estimate 95% CI Lower 95% CI			Upper	p-value
ACME (control)	0.001077	0.000797	0	<0.0000000000000002
ACME (treated)	0.001076	0.000796	0	<0.0000000000000002
ADE (control)	-0.001467	-0.003368	0	0.096
ADE (treated)	-0.001468	-0.003369	0	0.096
Total Effect	-0.000392	-0.002257	0	0.652
Prop. Mediated (control)	-2.749517	-17.709057	15	0.652
Prop. Mediated (treated)	-2.747628	-17.697553	15	0.652
ACME (average)	0.001076	0.000796	0	<0.0000000000000002
ADE (average)	-0.001468	-0.003369	0	0.096
Prop. Mediated (average)	-2.748572	-17.703305	15	0.652

#### 3.5.1 Female total score, depression, and cognition

In the mediation analysis focusing on female total score, depression, and cognitive impairment, we observed a significant indirect effect of female total score on cognitive impairment through depression. The estimates for the control and treatment groups were 0.001548 and 0.001550, respectively (95% CI: 0.001131 to 0.00 and 0.001134 to 0.00, p < 0.01). The average ACME was similarly significant (0.001549, 95% CI: 0.001133 to 0.00, p < 0.01).However, the direct effect was not statistically significant (mean ADE = 0.001281, 95% CI: -0.001395 to 0.00, p = 0.35), indicating that the influence of female total score on cognitive impairment arises primarily through depression. By contrast, the total effect attained statistical significance (estimate = 0.002829, 95% CI: 0.000168 to 0.01, p = 0.04), and the participation effect was likewise significant (mean estimate = 0.547415, 95% CI: 0.230549 to 2.79, p = 0.04). These observations imply that depression constitutes a key conduit by which female total score translates into increased cognitive impairment. Future inquiries may delve deeper into the mechanistic underpinnings of this relationship. For more information, see **Table 3**.

**Table 3:** Mediation Analysis of female Caregiver's Total Score and cognitive impairment with depression as a Mediator.

Estimate 95% CI Lower 95% CI			Upper	p-value
ACME (control)	0.001548	0.001131	0.00	<0.0000000000000002 ***
ACME (treated)	0.001550	0.001134	0.00	<0.0000000000000002 ***
ADE (control)	0.001280	-0.001394	0.00	0.04 *
ADE (treated)	0.001281	-0.003369	0.00	0.04 *
Total Effect	0.002829	0.000168	0.01	0.04 *
Prop. Mediated (control)	0.547125	0.230034	2.79	<0.0000000000000002 ***
Prop. Mediated (treated)	0.547705	0.231064	2.79	0.652
ACME (average)	0.001549	0.001133	0.00	<0.0000000000000002
ADE (average)	0.001281	-0.001395	0.00	0.35
Prop. Mediated (average)	0.547415	0.230549	2.79	0.04 *

* indicates significance at p < 0.05.

***indicates significance at p < 0.001.

#### 3.5.2 Male total score, depression, and cognition

When examining male total score, depression, and cognitive impairment, the indirect effect of male total score via depression was significant, with both the control group and treatment group estimates hovering around 0.00171 (95% CI: 0.00113 to 0.00, p < 0.01). The average ACME was also significant (0.00171, 95% CI: 0.00113 to 0.00, p < 0.0000000000000002), highlighting depression’s central mediating influence between male total score and cognitive impairment. Moreover, the direct effect of male total score on cognitive impairment was significant (mean ADE = -0.00922, 95% CI: -0.01311 to -0.01, p < 0.01), as was the total effect (estimate = -0.00750, 95% CI: -0.01145 to 0.00, p < 0.0000000000000002). The mediation path itself also reached a comparable level of significance (mean estimate = -0.22828, 95% CI: -0.52233 to -0.12, p < 0.0000000000000002), further underscoring depression’s integral part in shaping this association. Additional insights can be found in **Table 4**.

**Table 4:** Mediation Analysis of male Caregiver's Total Score and cognitive impairment with depression as a Mediator.

Estimate 95% CI Lower 95% CI			Upper	p-value
ACME (control)	0.00171	0.00113	0.00	<0.0000000000000002 ***
ACME (treated)	0.00171	0.00113	0.00	<0.0000000000000002 ***
ADE (control)	-0.00922	-0.01311	-0.01	<0.0000000000000002 ***
ADE (treated)	-0.00922	-0.01311	-0.01	<0.0000000000000002 ***
Total Effect	-0.00750	-0.01145	0.00	<0.0000000000000002 ***
Prop. Mediated (control)	-0.22830	-0.52271	-0.12	<0.0000000000000002 ***
Prop. Mediated (treated)	-0.22825	-0.52194	-0.12	<0.0000000000000002 ***
ACME (average)	0.00171	0.00113	0.00	<0.0000000000000002 ***
ADE (average)	-0.00922	-0.01311	-0.01	<0.0000000000000002 ***
Prop. Mediated (average)	-0.22828	-0.52233	-0.12	<0.0000000000000002 ***

***indicates significance at p < 0.001.

### 3.6 Adjusted analyses: total score and depression–dementia

After accounting for age, gender, marital status, educational level, place of residence, ethnicity, smoking habits, alcohol consumption, hypertension, diabetes, BMI, and obesity, we found that the total effect of total_score on dementia stood at approximately 0.001952 (p = 0.008). Though modest, this effect was still statistically significant in a large sample (N = 10,828). Depression’s average causal mediation effect was about 0.000394 (with an extremely low p), comprising roughly 20% of the total effect (Prop. Mediated ≈ 0.20, p = 0.008). This suggests that depression partially mediates the relationship between total_score and dementia, whereas the remaining 80% reflects direct effects (ADE ≈ 0.001558, p = 0.033) or other mechanisms. In essence, as total_score increases, approximately one-fifth of the additional dementia risk can be attributed to depression’s intermediary role. This underscores the potential value in managing depression among individuals with higher total scores. See **Table 5** for details.

**Table 5:** Mediation Analysis of Caregiver's Total Score and cognitive impairment with depression as a Mediator after adjusting for covariates.

Estimate 95% CI Lower 95% CI			Upper	p-value
ACME (control)	0.000393	0.000252	0.00	<0.0000000000000002 ***
ACME (treated)	0.000394	0.000253	0.00	<0.0000000000000002 ***
ADE (control)	0.001558	0.000129	0.00	0.033 *
ADE (treated)	0.001559	0.000129	0.00	0.033 *
Total Effect	0.001952	0.000531	0.00	0.008 **
Prop. Mediated (control)	0.201580	0.099109	0.68	0.008 **
Prop. Mediated (treated)	0.201818	0.099368	0.68	0.008 **
ACME (average)	0.000394	0.000252	0.00	<0.0000000000000002 ***
ADE (average)	0.001558	0.000129	0.00	0.033 *
Prop. Mediated (average)	0.201699	0.099238	0.68	0.008 **

* indicates significance at p < 0.05.

** indicates significance at p < 0.01.

***indicates significance at p < 0.001.

#### 3.6.1 Adjusted analyses: female caregiver total score and depression–dementia

Next, we examined the relationship among female_total_score, depression, and dementia under the same set of covariates. The model revealed that the total effect of the independent variable on the outcome, while relatively small (about 0.00456), was statistically significant (p = 0.0004). Further mediation analysis (ACME ≈ 0.000525, p nearly 0) indicated that depression serves as a partial mediator, accounting for around 11.5% of the effect (Prop. Mediated ≈ 0.115), with the remaining 88.5% attributable to the direct effect (ADE ≈ 0.004032). Although these effect sizes are not large, the extensive sample size confers statistical significance. Hence, in the linkage between female_total_score and dementia risk, depression functions as a critical, though partial, explanatory pathway. Future practical applications may include targeted clinical or public health interventions, as well as the exploration of additional mediators or interactions. More information is available in **Table 6**.

**Table 6:** Mediation Analysis of female Caregiver's Total Score and cognitive impairment with depression as a Mediator after adjusting for covariates.

Estimate 95% CI Lower 95% CI			Upper	p-value
ACME (control)	0.000524	0.000325	0.00	<0.0000000000000002 ***
ACME (treated)	0.000526	0.000326	0.00	<0.0000000000000002 ***
ADE (control)	0.004031	0.001932	0.01	0.0004 ***
ADE (treated)	0.004033	0.001933	0.01	0.0004 ***
Total Effect	0.004556	0.002471	0.01	0.0004 ***
Prop. Mediated (control)	0.114989	0.065585	0.23	0.0004 ***
Prop. Mediated (treated)	0.115381	0.065873	0.23	0.0004 ***
ACME (average)	0.000525	0.000326	0.00	<0.0000000000000002 ***
ADE (average)	0.004032	0.001933	0.01	0.0004 ***
Prop. Mediated (average)	0.115185	0.065729	0.23	0.0004 ***

***indicates significance at p < 0.001.

#### 3.6.2 Adjusted analyses: male caregiver total score and depression–dementia

Finally, we scrutinized the interplay among male_total_score, depression, and dementia, again controlling for the aforementioned covariates. The mediation analysis indicated that the total effect of the independent variable on dementia was extremely small and statistically non-significant (approximately -0.00031, p = 0.83), and the direct effect showed no significance either. Although the mediation effect (about 0.00076) had an exceptionally low p-value, it was negligible in real-world terms, and the proportion mediated was negative and non-significant. Consequently, these results imply that no discernible mediating mechanism exists for depression between male_total_score and dementia. Even with comprehensive adjustments for potential confounders, the association between male_total_score and dementia remains near zero, and depression’s mediating influence offers no robust explanatory value. See **Table 7** for further details.

**Table 7:** Mediation Analysis of male Caregiver's Total Score and cognitive impairment with depression as a Mediator after adjusting for covariates.

Estimate 95% CI Lower 95% CI			Upper	p-value
ACME (control)	0.000757	0.000467	0.00	<0.0000000000000002 ***
ACME (treated)	0.000757	0.000467	0.00	<0.0000000000000002 ***
ADE (control)	-0.001068	-0.004195	0.00	0.48
ADE (treated)	-0.001069	-0.004196	0.00	0.48
Total Effect	-0.000311	-0.003426	0.00	0.83
Prop. Mediated (control)	-2.431349	-9.247411	7.09	0.83
Prop. Mediated (treated)	-2.430051	-9.242343	7.09	0.83
ACME (average)	0.000757	0.000467	0.00	<0.0000000000000002 ***
ADE (average)	-0.001068	-0.004195	0.00	0.48
Prop. Mediated (average)	-2.430700	-9.244877	7.09	0.83

***indicates significance at p < 0.001.

## 4 Discussion

Prolonged exposure to stress—whether during fetal development, infancy, childhood, adolescence, adulthood, or old age—can have a profound impact on the brain structures integral to cognition and mental health (20). Various environmental stressors, including parent–child dynamics, strict or lenient disciplinary practices, and sibling favoritism, can trigger epigenetic processes that alter the expression of stress-related genes, thereby contributing to depression and cognitive impairment (21). Prior investigations have underscored a robust link between childhood stressors and both late-life depression and cognitive dysfunction (22–24). However, the bulk of existing research emphasizes the role of childhood adversity in later depression and cognitive decline, leaving comparatively scant attention to the more nuanced effects of parent–child relationships on depression and cognition during childhood. Against this backdrop, the present study capitalizes on a large dataset of 10,828 participants to illuminate the interplay among childhood caregiver relationships—encompassing relationships with parents, the degree of disciplinary strictness, and sibling favoritism—depression, and cognitive impairment. It further conducts an in-depth exploration of potential mediation pathways, offering new insights into how early-life familial environments might shape mental health trajectories.

Overall, our findings demonstrate marked discrepancies in a range of factors—such as gender, age, educational attainment, hypertension, depression, and parent–child (or caregiver) relationships—when comparing individuals with cognitive impairment to those with intact cognition. These results suggest that the aforementioned variables may bear a close association with cognitive function in later life. Notably, age emerges as a principal determinant of cognitive decline: as chronological age advances, so too does the risk of cognitive impairment (25). In parallel, lower educational levels are linked to heightened odds of cognitive dysfunction, whereas prolonged schooling can attenuate the deleterious effects of aging on cognition (26). Women also constitute a particularly vulnerable group, given that approximately two-thirds of Alzheimer’s patients are female (27). Our study found that the smoking rate in the cognitive impairment group was significantly lower than that in the cognitively normal group. Additionally, the proportion of alcohol drinkers in the cognitive impairment group was lower, with a notable increase in the difference in alcohol consumption levels. The relationship between smoking, alcohol consumption, and cognition remains controversial. Studies have shown that smoking and drinking often lead to cognitive decline (28).Our study found that the smoking rate in the cognitive impairment group was significantly lower than that in the cognitively normal group. Additionally, the proportion of alcohol drinkers in the cognitive impairment group was lower, with a notable increase in the difference in alcohol consumption levels. The relationship between smoking, alcohol consumption, and cognition remains controversial. Studies have shown that smoking and drinking often lead to cognitive decline (29).The relationship between alcohol consumption and cognitive impairment appears even more complex. Moderate alcohol consumption may have protective effects on cognition. For instance, Tiia Anttila and colleagues studied 1,018 participants and measured the prevalence of mild cognitive impairment and dementia in older adults. They found that individuals who abstained from alcohol or consumed alcohol frequently during middle age were twice as likely to develop mild cognitive impairment in later life compared to those who consumed alcohol occasionally. This suggests that the relationship between alcohol consumption and cognitive impairment is intricate and may depend on factors such as the amount and type of alcohol consumed (30, 31).Regarding body weight and obesity, the cognitive impairment group generally had lower body weight and obesity rates. However, studies have shown a strong connection between obesity and cognitive impairment, with obesity contributing to cognitive decline (32).Conversely, some studies have found that weight loss may accompany cognitive decline. For example, Jinlei Li and colleagues conducted a study on 3,632 participants from the Framingham Offspring cohort. They found that for individuals aged 40–49, an increase of one unit in BMI was associated with an elevated risk of dementia. However, after the age of 70, this risk appeared to decrease (33, 34).

Subsequent regression and subgroup analyses revealed that “total_score” and “female_total_score” each displayed a significant positive correlation with cognitive impairment. Conversely, “male_total_score” ceased to be significant after adjustment for covariates. This pattern implies that strained relationships with one’s parents are associated with a greater likelihood of cognitive deficits, and that depression consistently exerts a robust, positive impact in all models, underscoring its role as a pivotal risk factor. Previous studies have documented pronounced links between relationships with parents, disciplinary strictness, sibling favoritism, and cognitive outcomes. However, our results underscore that deteriorating relationships specifically with female caregivers raise the risk of cognitive impairment, whereas no analogous relationship was observed with male caregivers. One plausible explanation lies in the child-rearing practices prevalent in China, where women traditionally devote more time and resources to raising children (8, 35, 36).Turning to our mediation analysis—which explored the pathway from total_score (including its female- and male-specific variants) through depression to cognitive impairment—we observed divergent trends. Even after controlling for various covariates, total_score retained a significant, positive effect on cognitive impairment, with approximately 20% of that effect mediated by depression. In other words, higher childhood caregiver-relationship scores correlate with elevated levels of depression in adulthood, thereby increasing the risk of dementia. Although this effect is modest, it is nonetheless statistically significant in our large sample, highlighting depression as a crucial mediating variable. When covariates were further adjusted, the relationship between female_total_score and cognitive impairment became more pronounced, and around 11.5% of this link was mediated by depression. By contrast, male_total_score was no longer a significant predictor after adjustment. Although the male mediation effect reached an exceptionally low p-value, its magnitude was negligible, and the proportion mediated was negative and nonsignificant. These findings imply that, within male populations, childhood relationships with parents (or caregivers) do not exert a clear influence on later cognitive outcomes through depression. Instead, other, as-yet-unexplored mechanisms may be at play.

These findings bear several critical implications for both public health and clinical practice. Foremost, bolstering the quality of childhood relationships with parents (or caregivers) while minimizing physical punishment and curbing gender-biased parenting behaviors (e.g., preferential treatment of sons) may confer durable protective effects against cognitive decline in later life. Moreover, the synergistic influence of childhood relationship scores and depression on cognitive outcomes proves especially salient among women, underscoring the necessity for early screening and targeted preventive interventions for depression in this population. Finally, from a multidisciplinary vantage point, care strategies for middle-aged and older adults should integrate not only chronic disease management but also robust mental health support. Although this study provides valuable insights into the relationship between depression and cognitive impairment, there are several limitations and flaws when conducting mediation analysis using cross-sectional data, especially regarding causal inference and mediation effect models. Firstly, the use of cross-sectional data prevents us from clearly revealing causal relationships, particularly in mediation analysis. Cross-sectional data typically only shows associations between variables, without establishing causal order. Therefore, while we hypothesize that depressive symptoms may influence cognitive impairment through mechanisms such as cognitive function, the absence of temporal order prevents us from ruling out the possibility of reverse causality. For example, depressive symptoms may not only precede cognitive impairment but could also be a consequence of cognitive impairment, or there might be a bidirectional causal relationship between the two. This ambiguity in causal relationships is especially critical in mediation analysis, as it affects the accuracy and interpretability of the mediation effect model. As pointed out by Cole and Maxwell and Maxwell and Cole, mediation tests in cross-sectional designs that fail to retain temporal order can lead to biased model fitting estimates (37–39).Secondly, despite using standardized scales for measurement, the predictive power of the model in the mediation analysis is somewhat limited. The small effect sizes and confidence intervals including suggest that the relationships between some variables and the outcomes in the model are weak, and the predictive ability is not strong. This may imply that the mediation effects we tested are relatively weak or not significant in the current data. Since cross-sectional designs cannot fully control causal order, the model’s fit and predictive power may be affected. Therefore, future studies should consider more rigorous experimental designs or adopt longitudinal data to enhance the reliability of causal inferences. Additionally, this study uses a retrospective design, relying on participants’ recollections of childhood experiences and life circumstances, which may introduce selective memory and reporting bias. These measurement errors could not only affect the accuracy of independent variables (such as depressive symptoms and cognitive impairment) but also the assessment of mediating variables, thereby impacting the reliability of the mediation effects. While we controlled for some known covariates (such as demographic characteristics and health status), unmeasured potential confounders (e.g., psychosocial factors or biological mechanisms) might have influenced the mediation path. For example, long-term social stress or genetic background might play an important role in the mediation between depressive symptoms and cognitive impairment, but these factors were not included in the study model, which could lead to an underestimation or overestimation of the mediation effects. Thirdly, the study did not fully consider the interaction between dynamic variables, which is crucial for understanding mediation effects. For instance, the complex relationships between sleep behavior, social support, and mental health could alter the mediation path between depressive symptoms and cognitive impairment. Specifically, variables like sleep problems and social support may not only directly impact depression and cognitive impairment but could also modify the relationship between depressive symptoms and cognitive function, altering the strength or direction of the mediation effect. Therefore, future research should incorporate more dynamic interaction factors in cross-sectional designs or adopt longitudinal designs to better capture these interactions and their influence on mediation effects. Moreover, this study did not thoroughly explore the specific nature of the caregiver-child relationship. While we mentioned factors such as “relationship with parents, strictness of discipline, and sibling preference,” further description of these relational details is lacking. For example, the number of children in the family, whether caregivers provide academic support, nutritional status, emotional expression, and the duration of parental preferences or strict discipline could all significantly impact the parent-child relationship. These unexplored factors provide more specific insights for our recommendations. Therefore, future research should further examine these dimensions to better understand and improve the quality of caregiver-child interactions. Lastly, although the measurement of caregiver relationships was standardized in this study, the validity and reliability of the relevant scales in the Chinese population remain limited. The cultural adaptation and psychometric properties of the measurement tools are critical factors for ensuring the validity of the mediation analysis results. Thus, future research should further validate the applicability of these scales in the Chinese population and explore more suitable scales to improve the accuracy of mediation effect analysis. In summary, although this study conducted mediation analysis using cross-sectional data, the limitations in data design and measurement prevent us from drawing conclusions with strong causal inference. Therefore, we recommend that future research adopt longitudinal designs or other experimental methods to clarify causal relationships between variables and overcome the biases associated with cross-sectional data. Additionally, combining multiple data collection methods (such as biomarkers and self-reported data) will help reduce measurement bias and improve the reliability of mediation effects. Furthermore, exploring interactions between dynamic variables and further validating the psychometric properties of scales will deepen our understanding of the mediation path between depressive symptoms and cognitive impairment and enhance the predictive power of the model.

## 5 Conclusion

This study reveals that early-life caregiver relationships exert a noteworthy mediating effect on cognitive impairment via depression. Additionally, variables such as gender, educational attainment, place of residence, and caregivers’ disciplinary practices and favoritism exhibit significant interplay in shaping the association between the total score and cognitive impairment. Notably, women’s cognitive functioning appears particularly vulnerable to the dual influences of childhood adversities and depression. These findings highlight the critical importance of early psychological support and targeted interventions—namely, fostering a nurturing family milieu in childhood, broadening access to educational opportunities, and providing tailored support for depression—as pivotal strategies to mitigate the risk of cognitive impairment. Looking ahead, future research should adopt longitudinal designs and employ multimodal analyses (e.g., biomarker assessments and advanced imaging techniques) to elucidate causal pathways, explore potential gender-specific differences, and delineate underlying mechanisms. Such endeavors would yield robust scientific evidence to inform personalized prevention and intervention strategies aimed at preserving cognitive health across the lifespan.

## Data Availability

The original contributions presented in the study are included in the article/**Supplementary Material**. Further inquiries can be directed to the corresponding authors.
