# Supplementary material for: An analysis of the mediating influence of depression on the association between early-life caregiver relationships and cognitive function: a cohort study based on the CHARLS database
Source: Front Psychiatry. 2025 Mar 4;16:1555336. doi: 10.3389/fpsyt.2025.1555336 (PMC11914091; doi:10.3389/fpsyt.2025.1555336)
Supplement: Supplementary file 1 [file Table1.docx]

## The Relationship with Parents

1. J1_a :How would you rate your relationship with your female guardian when you were growing up?
2. Excellent
3. Very good
4. Good
5. Fair
6. Poor

2.J3_a How much love and affection did your female guardian give you while you were growing up?

1. Often

2. Sometimes

3. Rarely

4. Never

3.J4_a How much effort did your female guardian put into watching over you?

1. A lot

2. Some

3. A little

4. Not at all

4.J5_a How strict was your female guardian with her rules for you?

1. Very strict

2. Somewhat strict

3. A little strict

4. Not at all strict

5.J6_a Did your female guardian treat your siblings better than you when you were growing up?

1. Very strict

2. Somewhat strict

3. A little strict

4. Not at all strict

6.J8_a Did your female guardian prefer boys to girls?

1. Very much

2. Somewhat

3. A little

4. Not at all

7.K1_a When you were growing up, did your female guardian ever hit you? Was that often,sometimes, rarely, or never?

1. Often

2. Sometimes

3. Rarely

4. Never

8.J1_b How would you rate your relationship with your male guardian when you were growing up?

1. Excellent

2. Very good

3. Good

4. Fair

5. Poor

9.J5_b How strict was your male guardian with his rules for you?

1. Very strict

2. Somewhat strict

3. A little strict

4. Not at all strict

10.J6_b Did your male guardian treat your siblings better than you when you were growing up?

1. Very strict

2. Somewhat strict

3. A little strict

4. Not at all strict

11.J8_b Did your male guardian prefer boys to girls?

1. Very much

2. Somewhat

3. A little

4. Not at all

12.K1_b When you were growing up, did your male guardian ever hit you? Was that often,sometimes, rarely, or never?

1. Often

2. Sometimes

3. Rarely

4. Never
