# Supplementary material for: An analysis of the mediating influence of depression on the association between early-life caregiver relationships and cognitive function: a cohort study based on the CHARLS database
Source: Front Psychiatry. 2025 Mar 4;16:1555336. doi: 10.3389/fpsyt.2025.1555336 (PMC11914091; doi:10.3389/fpsyt.2025.1555336)
Supplement: Supplementary file 2 [file Table2.docx]

Stable1：Parent-child relationship total score and subgroup analysis of dementia

|  |  |  |  |
| --- | --- | --- | --- |
| **character** | **95% CI** | **p** | **p for interaction** |
| **sex** |  |  | 0.078 |
| female | 0.999(0.991,1.008) | 0.85 |  |
| male | 1.014(1.000,1.028) | 0.051 |  |
| **marital_status** |  |  | 0.103 |
| married | 0.999(0.992,1.007) | 0.825 |  |
| no married | 0.949(0.890,1.009) | 0.104 |  |
| **education** |  |  | 0.001 |
| illiterate | 1.021(1.004,1.039) | **0.017** |  |
| primary school | 1.007(0.995,1.019) | 0.265 |  |
| middle school | 0.966(0.943,0.989) | **0.004** |  |
| university | 1.067(0.966,1.174) | 0.192 |  |
| **location** |  |  | 0.022 |
| rural | 1.008(0.999,1.017) | 0.078 |  |
| urban | 0.990(0.977,1.003) | 0.118 |  |
| **nationality** |  |  | 0.47 |
| han | 0.999(0.992,1.007) | 0.863 |  |
| minority | 0.989(0.962,1.017) | 0.427 |  |
| **smoke** |  |  | 0.112 |
| no smoke | 0.996(0.988,1.004) | 0.381 |  |
| smoke | 1.012(0.995,1.029) | 0.179 |  |
| **drink** |  |  | 0.046 |
| no | 0.994(0.986,1.002) | 0.172 |  |
| <1m | 1.019(0.996,1.043) | 0.107 |  |
| >1m | 1.015(0.993,1.038) | 0.184 |  |
| **hypertension** |  |  | 0.006 |
| no | 1.007(0.998,1.016) | 0.154 |  |
| yes | 0.985(0.973,0.998) | **0.02** |  |
| **DM** |  |  | 0.339 |
| no | 1.000(0.992,1.008) | 0.995 |  |
| yes | 0.990(0.972,1.009) | 0.299 |  |
| **obesity** |  |  | 0.509 |
| over weight | 0.994(0.981,1.007) | 0.344 |  |
| normal | 1.004(0.993,1.014) | 0.479 |  |
| low weight | 0.994(0.963,1.026) | 0.699 |  |
| obesity | 0.990(0.969,1.010) | 0.32 |  |

Stable2：female_total score and subgroup analysis of dementia

|  |  |  |  |
| --- | --- | --- | --- |
| **character** | **95% CI** | **p** | **p for interaction** |
| **sex** |  |  | 0.188 |
| female | 1.009(0.996,1.022) | 0.161 |  |
| male | 1.027(1.004,1.049) | **0.019** |  |
| **marital_status** |  |  | 0.168 |
| married | 1.013(1.002,1.024) | **0.024** |  |
| no married | 0.947(0.856,1.041) | 0.268 |  |
| **education** |  |  | 0.001 |
| illiterate | 1.043(1.016,1.072) | **0.002** |  |
| primary school | 1.026(1.008,1.044) | **0.005** |  |
| middle school | 0.959(0.925,0.994) | **0.023** |  |
| university | 1.069(0.925,1.215) | 0.333 |  |
| **location** |  |  | 0.003 |
| rural | 1.032(1.018,1.046) | **<0.0001** |  |
| urban | 0.995(0.976,1.015) | 0.629 |  |
| **nationality** |  |  | 0.238 |
| han | 1.014(1.002,1.026) | **0.017** |  |
| minority | 0.987(0.945,1.030) | 0.559 |  |
| **smoke** |  |  | 0.104 |
| no smoke | 1.008(0.996,1.020) | 0.207 |  |
| smoke | 1.032(1.006,1.059) | **0.017** |  |
| **drink** |  |  | 0.211 |
| no | 1.007(0.995,1.020) | 0.24 |  |
| <1m | 1.037(1.001,1.074) | **0.045** |  |
| >1m | 1.028(0.993,1.064) | 0.117 |  |
| **hypertension** |  |  | 0.021 |
| no | 1.022(1.008,1.036) | **0.001** |  |
| yes | 0.994(0.976,1.014) | 0.568 |  |
| **DM** |  |  | 0.26 |
| no | 1.015(1.003,1.027) | **0.017** |  |
| yes | 0.997(0.970,1.025) | 0.854 |  |
| **obesity** |  |  | 0.279 |
| over weight | 1.001(0.981,1.021) | 0.91 |  |
| normal | 1.020(1.004,1.036) | **0.013** |  |
| low weight | 1.033(0.985,1.085) | 0.186 |  |
| obesity | 0.996(0.965,1.027) | 0.798 |  |

Stable3：male_total score and subgroup analysis of dementia

|  |  |  |  |
| --- | --- | --- | --- |
| **character** | **95% CI** | **p** | **p for interaction** |
| **sex** |  |  | 0.037 |
| female | 0.977(0.959,0.996) | **0.016** |  |
| male | 1.014(0.985,1.044) | 0.348 |  |
| **marital_status** |  |  | 0.146 |
| married | 0.972(0.957,0.987) | **<0.001** |  |
| no married | 0.886(0.778,1.002) | 0.06 |  |
| **education** |  |  | 0.017 |
| illiterate | 1.014(0.979,1.052) | 0.437 |  |
| primary school | 0.981(0.957,1.005) | 0.123 |  |
| middle school | 0.927(0.881,0.975) | **0.004** |  |
| university | 1.121(0.929,1.334) | 0.21 |  |
| **location** |  |  | 0.37 |
| rural | 0.978(0.960,0.997) | **0.021** |  |
| urban | 0.963(0.936,0.990) | **0.009** |  |
| **nationality** |  |  | 0.969 |
| han | 0.971(0.955,0.986) | **<0.001** |  |
| minority | 0.972(0.914,1.033) | 0.359 |  |
| **smoke** |  |  | 0.245 |
| no smoke | 0.969(0.952,0.986) | **<0.001** |  |
| smoke | 0.992(0.957,1.028) | 0.65 |  |
| **drink** |  |  | 0.014 |
| no | 0.960(0.943,0.977) | **<0.0001** |  |
| <1m | 1.014(0.967,1.063) | 0.561 |  |
| >1m | 1.014(0.970,1.061) | 0.533 |  |
| **hypertension** |  |  | 0.017 |
| no | 0.986(0.967,1.005) | 0.144 |  |
| yes | 0.948(0.923,0.972) | **<0.0001** |  |
| **DM** |  |  | 0.632 |
| no | 0.972(0.956,0.989) | **<0.001** |  |
| yes | 0.962(0.925,1.000) | 0.052 |  |
| **obesity** |  |  | 0.365 |
| over weight | 0.969(0.943,0.997) | **0.03** |  |
| normal | 0.978(0.957,1.000) | **0.046** |  |
| low weight | 0.921(0.864,0.981) | **0.011** |  |
| obesity | 0.963(0.922,1.005) | 0.088 |  |

Stable4：depression and subgroup analysis of dementia

|  |  |  |  |
| --- | --- | --- | --- |
| **character** | **95% CI** | **p** | **p for interaction** |
| **sex** |  |  | 0.355 |
| female | 1.762(1.604,1.937) | **<0.0001** |  |
| male | 1.621(1.394,1.883) | **<0.0001** |  |
| **marital_status** |  |  | 0.874 |
| married | 1.828(1.689,1.979) | **<0.0001** |  |
| no married | 1.730(0.881,3.431) | 0.113 |  |
| **education** |  |  | 0.144 |
| illiterate | 1.684(1.386,2.051) | **<0.0001** |  |
| primary school | 1.337(1.183,1.510) | **<0.0001** |  |
| middle school | 1.478(1.161,1.875) | **0.001** |  |
| university | 2.652(0.958,6.795) | **0.047** |  |
| **location** |  |  | 0.37 |
| rural | 1.672(1.520,1.839) | **<0.0001** |  |
| urban | 1.812(1.563,2.100) | **<0.0001** |  |
| **nationality** |  |  | 0.251 |
| han | 1.804(1.662,1.958) | **<0.0001** |  |
| minority | 2.157(1.608,2.899) | **<0.0001** |  |
| **smoke** |  |  | 0.133 |
| no smoke | 1.867(1.710,2.039) | **<0.0001** |  |
| smoke | 1.602(1.338,1.917) | **<0.0001** |  |
| **drink** |  |  | 0.645 |
| no | 1.814(1.660,1.982) | **<0.0001** |  |
| <1m | 2.019(1.588,2.569) | **<0.0001** |  |
| >1m | 1.729(1.351,2.215) | **<0.0001** |  |
| **hypertension** |  |  | 0.859 |
| no | 1.836(1.666,2.024) | **<0.0001** |  |
| yes | 1.809(1.580,2.071) | **<0.0001** |  |
| **DM** |  |  | 0.7 |
| no | 1.839(1.689,2.002) | **<0.0001** |  |
| yes | 1.759(1.427,2.169) | **<0.0001** |  |
| **obesity** |  |  | 0.061 |
| over weight | 1.884(1.635,2.172) | **<0.0001** |  |
| normal | 1.633(1.460,1.826) | **<0.0001** |  |
| low weight | 2.549(1.820,3.586) | **<0.0001** |  |
| obesity | 1.837(1.472,2.293) | **<0.0001** |  |
